# Imaging phonon dynamics with ultrafast electron microscopy: Kinematical and dynamical simulations

**DOI:** 10.1063/1.5144682

**Published:** 2020-04-17

**Authors:** Daniel X. Du, David J. Flannigan

**Affiliations:** Department of Chemical Engineering and Materials Science, University of Minnesota, 421 Washington Avenue SE, Minneapolis, Minnesota 55455, USA

## Abstract

Ultrafast x-ray and electron scattering techniques have proven to be useful for probing the transient elastic lattice deformations associated with photoexcited coherent acoustic phonons. Indeed, femtosecond electron imaging using an ultrafast electron microscope (UEM) has been used to directly image the influence of nanoscale structural and morphological discontinuities on the emergence, propagation, dispersion, and decay behaviors in a variety of materials. Here, we describe our progress toward the development of methods ultimately aimed at quantifying acoustic-phonon properties from real-space UEM images via conventional image simulation methods extended to the associated strain-wave lattice deformation symmetries and extents. Using a model system consisting of pristine single-crystal Ge and a single, symmetric Lamb-type guided-wave mode, we calculate the transient strain profiles excited in a wedge specimen and then apply both kinematical- and dynamical-scattering methods to simulate the resulting UEM bright-field images. While measurable contrast strengths arising from the phonon wavetrains are found for optimally oriented specimens using both approaches, incorporation of dynamical scattering effects via a multi-slice method returns better qualitative agreement with experimental observations. Contrast strengths arising solely from phonon-induced local lattice deformations are increased by nearly an order of magnitude when incorporating multiple electron scattering effects. We also explicitly demonstrate the effects of changes in global specimen orientation on the observed contrast strength, and we discuss the implications for increasing the sophistication of the model with respect to quantification of phonon properties from UEM images.

## INTRODUCTION

Photoexcitation of materials below the ablation threshold with ultrashort laser pulses leads to the generation of coherent acoustic phonons composed of a propagating elastic lattice distortion.^1–4^ The precise nature and the behavior of these transient elastic deformations depend upon a number of factors, including the structure, composition, and morphology, as well as specimen geometry, defect density and type, and photoexcitation conditions.^5–10^ As such, the observed responses can be varied and complex, with the spatiotemporal dynamics evolving over times ranging from femtoseconds to microseconds or longer. Nevertheless, significant advances in understanding have been made, most often by employing various ultrafast spectroscopic techniques, such as time-domain Brillouin and Raman scattering and ultrafast optical reflectivity.^11,12^ In general, these approaches employ all-optical femtosecond (fs) laser pulses based on a conventional pump-probe methodology. In this way, the properties of impulsively excited strain-wave dynamics can be probed with up to combined nanometer-picosecond spatiotemporal resolutions, thus providing access to transient elastic responses associated with low-frequency, coherent acoustic phonons.

As acoustic-phonon dynamics produce a transient structural perturbation, in essence meaning the atoms comprising the material are set in motion, such behaviors are especially amenable to study using fs x-ray scattering and fs electron scattering techniques. Ultrafast diffraction and reflectivity experiments employing a large probe size relative to lattice dimensions have been used extensively, where transient variations in the properties of coherently scattered x-ray photons or fast electrons in the form of Bragg beams, or variations in transmitted- or reflected-beam intensities, are caused by the elastic strain waves.^13–33^ The advent of fs electron microscopes (i.e., ultrafast electron microscopy, UEM) has led to the application of ultrafast selected-area and convergent-beam diffraction (CBED) and ultrafast real-space imaging to the study of acoustic-phonon dynamics.^34–40^ Compared to ultrafast diffraction with essentially plane wave illumination, ultrafast CBED makes use of a convergent, typically nanometer-sized focused beam, as in conventional CBED (though ultrafast convergent microdiffraction and convergent-beam ultrafast electron crystallography are also used in dedicated diffraction instruments).^24,39,41^ In this way, discrete nanoscale three-dimensional (i.e., multiple Laue zones) crystallographic structural dynamics can be studied.^42–44^ Furthermore, positioning and rastering of the beam in ultrafast CBED allow one to spatiotemporally map transient strain profiles in selected locations relative to discrete lattice discontinuities, such as vacuum–crystal interfaces.^45^

Ultrafast imaging using a UEM also provides useful capabilities that are complementary to ultrafast diffraction measurements in that spatiotemporally distinct responses across a relatively large field of view, and in three spatial dimensions, can be captured in as few as one image series.^46–51^ Accordingly, disparate dynamics influenced by individual lattice discontinuities, as well as spatiotemporally varying phonon dispersion and relaxation behaviors (i.e., scattering and interference), can be directly surveyed relatively efficiently.^52–57^ For example, UEM bright- and dark-field imaging has been used to observe the generation of coherent, hypersonic contrast-wave dynamics in single-crystal Ge.^52,58^ By tracking individual wavefronts and mapping the time-varying phase-velocity dispersion of the contrast waves, the associated transient strain states were identified as arising from excitation of a single, first-order symmetric (*S*_1_) Lamb-type guided-wave mode. Furthermore, UEM imaging has been successfully used to resolve the influence of individual lattice discontinuities in the form of multilayer terraces and few-molecular-layer step-edges on spatially discrete phonon dynamics, with spatially resolved picosecond differences in oscillatory responses of neighboring bend contours revealing the position and the size of the defect.^51,52,54^ Indeed, in combination with ultrafast diffraction and ultrafast electron energy-loss spectroscopy,^59–61^ UEM imaging provides a means to generate a comprehensive understanding of photoinduced elastic structural dynamics spanning many orders of magnitude in combined space, energy, and time.

Qualitative interpretations of the observed UEM-image contrast behaviors arising from strain-wave dynamics typically invoke physical pictures of transient structural distortions analogous to bend contours in TEM imaging and modulation of the excitation error, *s***_*g*_**, in reciprocal space (i.e., the extent of intersection of the Ewald sphere with the relrods, where ***g*** is the scattering vector).^51,54,55,62,63^ This reciprocal-space perspective follows from the kinematic approximation, a key aspect of which provides for only a single, spatially averaged scattering event per incident electron.^64,65^ The kinematic approximation is extended and further developed into the dynamical model by including additional scattering events as the electron propagates through the material. Indeed, inclusion of dynamical effects is often necessary for achieving accurate quantitative descriptions owing to the large scattering cross sections relative to x-ray photons. Methods to simulate such phenomena in the context of ultrafast x-ray diffraction have been developed, and it has been shown that dynamical scattering must be considered in order to accurately quantify and interpret ultrafast electron diffraction patterns.^66–69^

Here, in order to make progress toward an accurate, quantitative picture of UEM imaging of propagating acoustic-phonon dynamics, we apply conventional descriptions of dynamical scattering in real space to simulations of a wedge-shaped specimen experiencing transient, propagating elastic deformations due to excitation of a Lamb-type guided wave. We first explicitly demonstrate how the kinematic approximation for the chosen conditions falls short of accurately describing the quantitative strain profiles before illustrating how inclusion of dynamical effects via multi-slice techniques is necessary for accurately capturing the scattering response. We largely follow the general approaches to simulating electron micrographs initially developed by Howie and Whelan and extended by others to the development of descriptions of contrast formation by discrete defects.^70–73^ As described below, we extend these approaches to UEM imaging on the relevant spatiotemporal scales on which acoustic phonons are active (i.e., nanometers and picoseconds), and we largely follow De Graef's formulations of the kinematical and dynamical models.^64^

## LAMB-MODE GUIDED-WAVE CONTINUUM MODEL

**FIG. 1.**
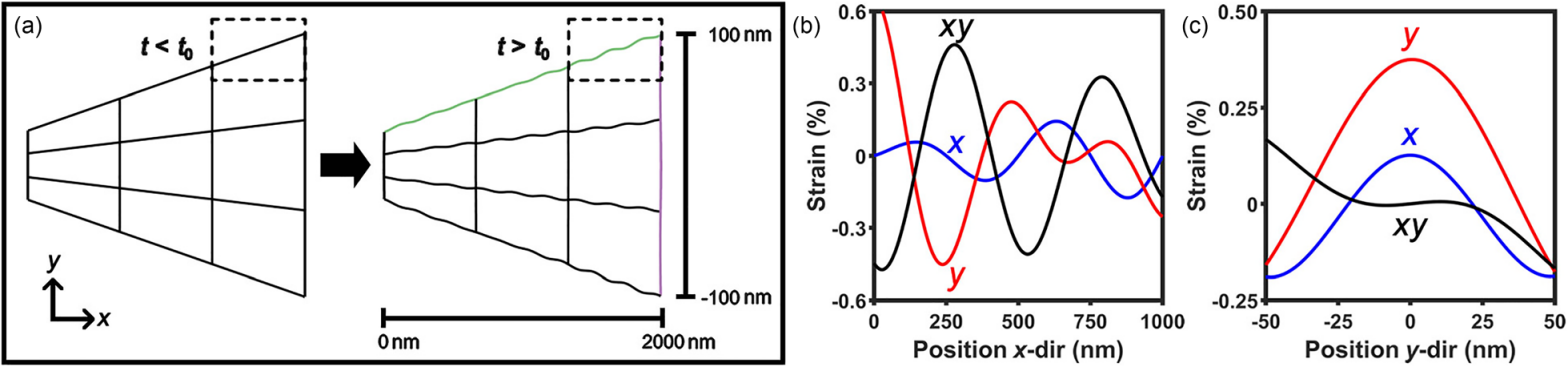
Depiction of the transient structural distortion and the resulting quantitative strain for the first-order symmetric Lamb mode (*S*_1_) in a photoexcited wedge specimen. (a) A wireframe, cross-sectional side view of the wedge. The wedges shown are before (left) and after (right) fs photoexcitation (i.e., *t* < *t*_o_ and *t* > *t*_o_, respectively, where *t*_o_ is when the peak of the Gaussian excitation pulse arrives at the specimen). The common regions in the dashed rectangles highlight the resulting distortion following photoexcitation. The distortion of the wireframe is greatly exaggerated for illustrative purposes. (b) The percent strain spatial profile in the *x* and *y* directions [according to the coordinate system in (a)] for the dilatational symmetry (blue and red, respectively) along the top surface of the wedge for *t* > *t*_o_ [green in panel (a)], in addition to the *xy* shear component (black). (c) The percent strain spatial profile in the *x* and *y* directions for the dilatational symmetry (blue and red, respectively) through the width of the wedge for *t* > *t*_o_ [purple in panel (a)], in addition to the *xy* shear component. The percent strain in panels (b) and (c) is on the order of the calculated strains arising from the *S*_1_ Lamb mode (see below).

The general architecture of the simulations consisted of a continuum model for the first-order symmetric guided-wave Lamb-mode distortion (*S*_1_) propagating within a structurally isotropic, compositionally pure, and defect-free wedge-shaped specimen. Here, we chose single-crystal Ge as the model system owing to its isotropic elastic properties and to the relative simplicity with which electron-scattering behaviors can be calculated. The underpinning fundamental behaviors modeled here follow the general descriptions of guided-wave behavior in isotropic solids,^74^ with the simulations focusing on translating theoretical physical phenomena to contrast-wave behaviors in fs electron imaging in UEM. The basic specimen geometry and orientation, as well as the spatially deconvoluted strain profiles resulting from the transient, propagating structural distortion of the simulated system, are shown in Fig. 1. A single Lamb mode consisting of wavefronts having a common velocity, a common frequency, and a common wavenumber was employed to reduce the computational cost and to place emphasis on quantitatively linking the transient strain profiles to UEM bright-field images. It should be noted that this is not entirely indicative of experimentally observed behaviors in such systems, where a time-varying phase-velocity dispersion of the *S*_1_ mode in Ge wedge specimens has been observed.^58^ Nevertheless, exclusion of this particular behavior did not impact the developed associations of the strain-wave profiles and the resulting image contrast, as the emphasis here was on simulating the distortion rather than the detailed time-varying behavior.

Specifications of the simulated specimen and the fundamental Lamb-mode distortions used to determine the transient strain profiles are as follows. The system prior to excitation consisted of an undistorted wedge specimen with a semi-angle of 1° and a leading-edge thickness of 50 nm [Fig. 1(a), left]. A perturbation in the form of an *S*_1_ Lamb mode for a plate of uniform 50-nm thickness (for simplicity) was then applied [Fig. 1(a), right], and the resulting longitudinal and transverse transient strain profiles were mapped in two dimensions [Figs. 1(b) and 1(c)]. For completeness, the *xy* shear profile was also mapped. The maximum simulated strain was on the order of 1%, as set by the chosen oscillation amplitude of 6 nm^2^ (see Table I). It is worth noting that extension of physical descriptions of static applied stresses may be deficient when describing the ultrafast, short-lived transient stresses associated with the propagating strain waves of interest here. Though not the focus, questions can be posed regarding timescales of dislocation motion, effective residence time of the propagating stress front, and the resulting onset of plasticity at larger strains.

**TABLE I.** Variables, definitions, and units used in the Lamb-mode guided-wave model.^58,75^

Variable	Definition	Value	Units
*V_L_*	Longitudinal speed of sound	5.350	nm ps^−1^
*V_T_*	Transverse speed of sound	3.570	nm ps^−1^
*V*	Phase velocity	8	nm ps^−1^
*h*	Half-thickness	25	nm
*A*	Amplitude	6	nm^2^
*k*	Wavenumber	0.0270	nm^−1^
*ω*	Frequency	35.740	GHz

The *x-* and *y*-axis distortions as a function of distance *y* from a line bisecting the wedge in the *x* direction [see Fig. 1(a)] are given by^74^

$$u=qA\left[\text{cos}\left(qy+\alpha \right)-\frac{2k^{2}}{k^{2}-q^{2}}\frac{\text{cos}\left(qh+\alpha \right)}{\text{cos}\left(ph+\alpha \right)}\text{cos}\left(py+\alpha \right)\right]\exp\left[i\left(kx-\omega t\right)\right],$$ (1)

$$v=kA\left[\text{sin}\left(qy+\alpha \right)+\frac{2pq}{k^{2}-q^{2}}\frac{\text{cos}\left(qh+\alpha \right)}{\text{cos}\left(ph+\alpha \right)}\text{sin}\left(py+\alpha \right)\right]\exp\left[i\left(kx-\omega t\right)\right],$$ (2)

$$p^{2}=\omega^{2}\left(\frac{1}{V_{L}^{2}}-\frac{1}{V^{2}}\right),$$ (3)

$$q^{2}=\omega^{2}\left(\frac{1}{V_{T}^{2}}-\frac{1}{V^{2}}\right).$$ (4)

Here, *A* is an amplitude term, *k* is the wavenumber, *h* is the characteristic thickness of the specific Lamb mode, and *ω* is the frequency of the wave. The parameters *V_L_*, *V_T_*, and *V* are the longitudinal, transverse, and phase velocities, respectively. The parameters *V_L_* and *V_T_* are material-dependent properties, while *k*, *ω*, and *V* are specific to the Lamb mode in question.^75^ As mentioned above, *A* was set such that the maximum elastic strain approached 1%. All other values for the oscillation parameters were matched to experimentally observed values.^58^ See Table I for the specific values used here. From these calculated distortions and the resulting strain profiles, simulations of bright-field images of the model system shown in Fig. 1(a) were performed using both kinematical and dynamical scattering models.

## KINEMATICAL SIMULATIONS

Kinematical simulations of strain-wave dynamics are rooted in calculation of the modulation of the excitation error, *s***_*g*_**, due to associated motion of the reciprocal lattice, and therefore motion of the relrods (in the case of a thin plate of uniform thickness, as approximated here), with respect to a fixed, single Ewald sphere for a common incident electron wave vector, ***k_i_***, and a single electron energy. Generally, because the specific relrod intensity distributions, sizes, and orientations are dictated by the real-space specimen geometry and orientation, motion of the specimen due to the propagating strain waves will cause local variations in *s***_*g*_** that are dictated by the symmetry of the mode and the precise moment in time for a particular spatial position. Accordingly, these variations must be calculated based on the properties of the relrod (as dictated by the specimen) and the time-varying intersection point of the relrod with the Ewald sphere (as dictated by the orientation of ***k_i_*** with respect to the specimen). This will return the transient scattering intensity of the Bragg beam associated with the scattering vector ***g***.

The intensity, *I***_*g*_**, of the Bragg beam can be found using the multi-beam kinematical expression shown as follows:^64^

$$I_{g}=\frac{{\text{sin}}^{2}\left(\pi s_{g}z_{0}\right)}{{\left(s_{g}\xi_{g}\right)}^{2}}e^{-\alpha z_{0}}.$$ (5)

Here, *s***_*g*_** is the excitation error of the relrod at scattering vector ***g*** (both in units of nm^−1^), *z*_0_ is the specimen thickness through which the electron propagates, and *ξ***_*g*_** is the extinction distance for scattering vector ***g***. Absorption by the specimen is given by the exponential decay term and is characterized by the absorption distance, *α*, in units of nm^−1^.^64,76^ An additional condition was imposed wherein each point in the specimen had a normalized group of intensities. That is, each real-space position had associated with it a vector of intensities corresponding to the Bragg spots. Thus, the magnitude of this vector, with each component multiplied against its complex conjugate, is equivalent to unity. This normalization is applied before the absorption exponential term, as each Bragg spot is monotonically affected by the same decay.^64^ While both bright-field and dark-field images can be simulated in this way, attention here is focused only on the bright-field results for simplicity. Note that only the intensity of the main beam was used at each point of interest in forming the images.

For the kinematic approximation, the excitation error, *s***_*g*_**, is the distance in reciprocal space between the actual intersection point of the Ewald sphere with the relrod and the geometric center of the relrod (i.e., the exact Bragg condition), as given by^64^

$$s_{g}=\frac{-g\cdot \left(2k_{i}+g\right)}{2\left|k_{i}+g\right|\text{cos}\beta }.$$ (6)

Here, *β* is the angle in radians between the real-space surface normal of the specimen and the direction of ***k_i_***. The extinction distance, *ξ***_*g*_**, is calculated via a Doyle-Turner-style parameterization using parameters from the study by Peng *et al.* for inelastic scattering properties at 273 K, as shown by the following equation:^76^

$$f^{\prime }\left[Å\right]\left(s\;\left[{Å}^{-1}\right]\right)=E_{F}\sum_{i=1}^{5}a_{i}\;\exp\left(-b_{i}s^{2}\right).$$ (7)

Here, *a* and *b* are the parameters of interest and have units of Å and Å^2^, respectively, and *i* is an index. See Table II for a listing of the specific parameter values for each index *i*. Note that because the objective here was to isolate the scattering caused by the *S*_1_ Lamb mode distortion and to initially build in complexity from the kinematical to the dynamical models, thermal contributions to signal intensity arising from the Debye-Waller effect were ignored. Thus, the terms for the Debye–Waller factor have been excluded from the equations presented here.

**TABLE II.** Values of the parameters for Ge for each index *i* for the kinematic approximation.^76^

Index, *i*	*a_i_* (Å)	*b_i_* (Å^2^)
1	−0.0008	0.0002
2	−0.0281	0.1071
3	0.1559	0.5417
4	0.0873	1.6461
5	0.0110	8.4617

The term *E_F_* outside the summation in Eq. (7) serves as a conversion factor between the scattering factors for 100-keV electrons and electrons of any other energy, here 200 keV. The conversion factor for these two energies is explicitly given by the following equation for convenience:

$$E_{F}=\frac{{\left[1-{\left(1+1.957\;9341\times 10^{-3}\cdot 100\;\mathrm{keV}\right)}^{-2}\right]}^{-1}}{{\left[1-{\left(1+1.9\;579\;341\times 10^{-3}\cdot 200\;\mathrm{keV}\right)}^{-2}\right]}^{-1}}.$$ (8)

Conversion to the Fourier coefficients of the electrostatic lattice potential is accomplished using the following equation:

$$V_{g}^{\prime }\left[V\right]=\frac{47.87\;801}{\Omega \;\left[{\mathrm{nm}}^{3}\right]}f^{\prime }\left(s\left[{Å}^{-1}\right]\right)F\left(\theta \right).$$ (9)

Here, Ω is the unit-cell volume in units of nm^3^, *F*(*θ*) is the structure factor at a specific reciprocal-space position, and *f ′*(*s*) is the aforementioned atomic absorption scattering factor. The value in the numerator of the ratio before the product of the absorption scattering and the structure factor comes from conversion of the Mott-Bethe formula, which relates x-ray scattering factors to electron scattering factors and to the Doyle–Turner parameterization. Equation (9) can be converted to a more convenient variable form via the following equation:^64^

$$U_{g}^{\prime }=\frac{2me}{\hbar^{2}}V_{g}^{\prime }.$$ (10)

Here, *m* is the relativistic electron mass, *e* is the fundamental charge, and *ℏ* is the reduced Planck constant. From this, the normal absorption length, which is monotonically applied across the diffraction pattern, determines the effect of electrons lost due to inelastic processes, as given by the following equation:

$$\frac{1}{\xi_{0}^{\prime }}=\frac{\left|U_{0}^{\prime }\right|}{\left|k_{i}\right|}.$$ (11)

Here, $\xi_{0}^{\prime }$ is the absorption length and $U_{0}^{\prime }$ is the imaginary part of the Fourier coefficients of the electrostatic lattice potential at the reciprocal-space origin. From this treatment and a normalization step, the final intensity [Eq. (12)] is found,

$$I=I_{g}\text{exp}\left(-\frac{2\pi }{\xi_{0}^{\prime }}z_{0}\right).$$ (12)

**FIG. 2.**
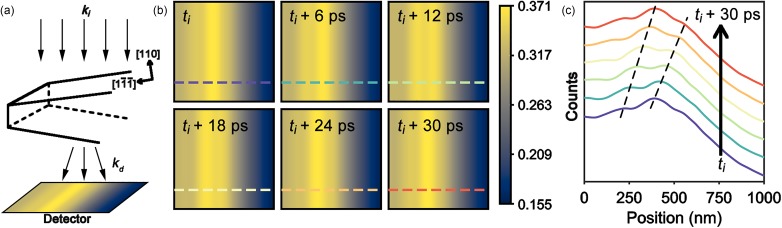
Simulated UEM bright-field images of transient, propagating distortions arising from excitation of the *S*_1_ Lamb mode using the kinematic approximation. (a) Schematic illustrating the general geometric arrangement of the incident electron wave vector, ***k_i_***, the wedge specimen, the diffracted electron wave vectors, ***k_d_***, and the detector. The wedge surface-normal is the [*110*] direction. An example false-colored, simulated image is shown on the detector. Multiple parallel arrows indicate the parallel-beam illumination condition and direction (i.e., a single ***k_i_***). (b) Series of simulated UEM images at times *t* after excitation of the distortion, where *t_i_* > *t*_o_ is set as the initial response, and all other images are relative to *t_i_* and occur at 6-ps increments. Note that the specimen is tilted to *α* = 5° and *β* = 2° with respect to ***k_i_***. See Fig. 4 for the tilt-angle orientations with respect to the specimen and ***k_i_***. The colored dashed lines indicate the positions from which the line profiles in (c) were generated. The color bar is in units of counts normalized to vacuum. (c) Line profiles generated from the simulated images in (b). The colors correspond to the colored dashed lines in (b). The profiles are offset for clarity, and the propagating nature of the waves is illustrated with the black dashed lines, each of which connects the peaks of a particular individual wavefront.

With the kinematic scattering condition set, the geometric arrangement of the Ge wedge specimen under the beam was configured next. The top surface of the wedge specimen was chosen to be the (*110*) plane, with the [$1\bar{1}\bar{1}$] direction pointing toward the leading edge [Fig. 2(a)]. The specimen was then tilted by an additional *α* = 5° and *β* = 2° with respect to ***k_i_*** (see Fig. 4 for tilt-angle orientations with respect to ***k_i_***). For image formation, a total of 400 beams surrounding the main beam out to a scattering angle of 3.1° were calculated to determine the fraction of electrons scattered away from the bright-field condition. Contributions by beams with angles larger than this were assumed to be negligible and were thus ignored. Furthermore, only allowed Bragg beams for the [*110*] zone axis were considered. Owing to the wedge geometry of the specimen, two relrods—one for each unique surface—were included for each reciprocal lattice position.^65^ For a specific time point, *t*, scattering intensity was calculated at positions separated by 10 unit cells along a 1-*μ*m length of the specimen [Figs. 2(b) and 2(c)]. This was repeated six select times, the temporal separation of which (Δ*t *=* *6 ps) was chosen to allow for illustration of the propagating nature of the strain wave for the conditions used here; each wave propagated 48 nm between simulated frames. At each 10-unit-cell step across the horizontal length of the image, the average strain encountered by the incident electron passing through the specimen was calculated in order to find the position of the scattering vector, ***g***, and therefore the excitation error, *s***_*g*_**. Following this, the total distance traveled by the electron, and thus the total intensity (i.e., counts), was determined for each vertical slice, and simulated images and line profiles were constructed.

As can be seen in Fig. 2, the simulated UEM bright-field images of Lamb-wave propagation using the kinematic approximation do indeed result in measurable contrast strengths, as experimentally observed.^52,58^ However, comparison of these results to experiment indicates the simulated kinematic contrast strengths are significantly weaker. Here, the maximum and minimum normalized counts differ by a factor of 2.4 [Fig. 2(b)]. Reasons for the discrepancy may stem from the inherent simplicity of the kinematic scattering approximation, the actual physical mechanisms giving rise to much larger experimentally realized strains without the onset of plasticity, additional non-strain effects contributing to the observed contrast strength, or a combination thereof. Though additional physical contributions to the experimentally observed contrast strengths may be present, and while ultrafast hyperelastic deformation may be at work, it is best to first increase the complexity of the scattering model in order to better model the contrast-forming mechanisms and resulting image profiles before invoking more exotic explanations. Consequently, a dynamical model, explored in the section on Dynamical Multi-Slice Simulations, was implemented in an attempt to better match the actual scattering conditions.

Prior to describing the simulations based on dynamical scattering, a note on the phonon wavefront velocity behavior illustrated in Fig. 2(c) is provided. While the simulations do capture the fixed velocity and single-propagation direction of each wavefront (indicated by the dashed line), an apparent dispersion behavior also emerges, as indicated by the differing slopes of the neighboring wavefronts. Though this matches experimental observations,^58^ it in fact arises from an obfuscation of the peak position of the contrast, rather than an actual physical response, owing to the use of a single, non-dispersive Lamb mode (see Table I). This is also apparent in the velocities that emerge in the dynamical-scattering simulations (see below). It is likely that the apparent dispersion is caused by a linear combination of the underlying intensity of the undistorted wedge with the distortion of the wave itself, which shifts the true position of the contrast peaks relative to the position of greatest strain. In addition, diffraction contrast will add complexities in the form of intensity fringes for certain specimen thicknesses and geometries. Thus, the observed phonon speed depends upon the overall curvature of the intensity modulation. That is, in cases where there is no curvature (i.e., where there is a constant slope in the background specimen intensity), the speed will be constant when observed at different locations in the wedge. However, if the non-distorted-wedge intensity curve is fluctuating, the perceived peak position of the contrast wave will spatially shift, thus resulting in an apparent altered speed. Ultimately, this will not impact the experimental observations so long as a single location is consistently analyzed, though artifacts may be introduced if the analysis position is varied without accounting for such effects by, for example, background subtraction and generation of difference images.

## DYNAMICAL MULTI-SLICE SIMULATIONS

The Lamb-mode guided-wave continuum model used for the kinematic approximation (described above) was also used for the dynamical scattering simulations. The dynamical scattering case is fundamentally different from the kinematic approximation in that incident electrons are no longer restricted to a single scattering event as they propagate through the specimen. Instead, multiple-scattering events are accounted for. Conveniently, the base equations used in the kinematic approximation [Eqs. (7)–(10)] are also applicable in dynamical scattering, the main difference being that differential equations are numerically integrated in a multi-slice method in order to account for multiple scattering.^64^ Values of the indices used in the parameterization for the elastic atomic scattering factors for the dynamical scattering approach are shown in Table III. Accordingly, the Howie–Whelan equations for dynamical scattering were employed here and were extended to the Lamb-mode elastic distortions propagating through a Ge wedge specimen.^77^ Note that the Howie–Whelan equations were used here instead of the more rigorous Bloch wave formalism in order to focus on the effects of a multi-slice method that aims to capture only the impact of multiple-scattering processes on the transient strain profiles.

**TABLE III.** Values of the parameters for Ge for each index *i* for dynamical scattering.^76^

Index, *i*	*a_i_* (Å)	*b_i_* (Å^2^)
1	0.213 5	0.098 9
2	0.976 1	0.984 5
3	1.655 5	4.552 7
4	2.893 8	21.556 3
5	1.635 6	70.390 3

The central differential equation governing electron scattering in dynamical theory is shown by the following equation:^64^

$$\frac{d\psi_{g}}{dz}=i\pi \left[2s_{g}\psi_{g}+\sum_{g^{\prime }}\frac{1}{\xi_{g-g^{\prime }}}\psi_{g^{\prime }}\right].$$ (13)

Here, $\psi_{g}$ is the wave function of the beam associated with scattering vector ***g***, *z* is the distance along the electron-propagation direction, *s***_*g*_** is the excitation error of scattering vector ***g***, and $\frac{1}{\xi_{g-g^{\prime }}}$ is an interaction parameter between the scattered beams determined by the Fourier coefficients of the electrostatic potential.^64^ As with the kinematic approximation, the elastic atomic scattering factors are again calculated from a Doyle-Turner-style parameterization with the parameters *a* and *b* sourced from Peng [Eq. (7) and Table III),^78^ and the Fourier coefficients of the electrostatic lattice potential are found using Eq. (9) and simplified using Eq. (10) in order to calculate the extinction distances.

For dynamical theory, the extinction distance is given by the following equation:

$$\frac{1}{\xi_{g\neq 0}}=\frac{\left|U_{g}\right|}{\left|k_{i}+g\right|},\;\frac{1}{\xi_{0}}=0.$$ (14)

Here, *U***_*g*_** is the real part of the Fourier coefficients of the electrostatic potential at any scattering vector, ***g***. Note that the column approximation was used here, where deviation from the initial electron propagation direction is assumed to be negligible.^79^ Simplification into a matrix form results in the following equation:^64^

$$\frac{d\psi }{dz}=i\pi A\psi .$$ (15)

Here, *A* is the matrix form of the right side of Eq. (13) and is given as Eq. (16), and *ψ* is the vector of root-intensities pertaining to each Bragg diffracted beam. The initial vector of *ψ* contains zeros at all positions except the first, which is set to 1,

$$A=\left[\begin{array}{ccc} 2s_{g_{1}} & \cdots & \frac{1}{\xi_{g_{N}-g_{1}^{\prime }}}\\ \vdots & \ddots & \vdots \\ \frac{1}{\xi_{g_{1}-g_{N}^{\prime }}} & \cdots & 2s_{g_{N}} \end{array}\right].$$ (16)

Here, $A_{nn}=2s_{g_{n}}$ and $A_{nn^{\prime }}=\frac{1}{\xi_{n-n^{\prime }}},\;n\neq n^{\prime }$.

Each slice in the direction of propagation was individually integrated. Again, the initial conditions were set such that the main beam, *ψ*_0_, was equal to one, while matrix positions associated with *ψ***_*g*_** were populated with a zero. Each individual slice was calculated every 1 Å through the specimen thickness.^72^ The result of a single step was found by initially finding the root-intensity change via a matrix exponential shown by the following equation:

Δψ=ψexp(iπΔzA). (17)

The total intensity, ${\left|\psi_{g}\right|}^{2}$, for a particular slice for the diffracted beam associated with scattering vector ***g*** was then normalized to one over all *N* beams. By integrating across the entire specimen thickness, the final solution then takes the following form:

$$I_{g}={\left|\psi_{g}\right|}^{2}e^{-\alpha z_{0}}.$$ (18)

The bright-field image is then constructed using the direct-beam intensity at each spatiotemporal point.

Note that at each slice, normalization similar to that for the kinematic approximation was carried out in order to conserve the electron count. That is, absorption is taken into account only after the electron exits the specimen, as the monotonic nature of absorption is unaffected by the multi-slice technique and is therefore readily factored out of the numerical integration. Also note that the approach described here, specifically with respect to stepwise integration along the electron propagation direction, is reasonable because the transient structural distortion occurs on a much longer timescale than that of the fast electron passing through the specimen (picoseconds compared to hundreds of attoseconds; the phonon wavefront propagates 4 pm in the time taken for a 200-keV electron to pass entirely through the specimen, much smaller than the 10-unit-cell lateral sampling size and 1-Å sampling size in the *z*-direction). Here, the electron-packet duration is set to the time it takes a single electron to pass through the specimen, while experimental durations are significantly longer and will need to be incorporated into more sophisticated models aiming to quantify phonon strain properties.^27,80–84^

**FIG. 3.**
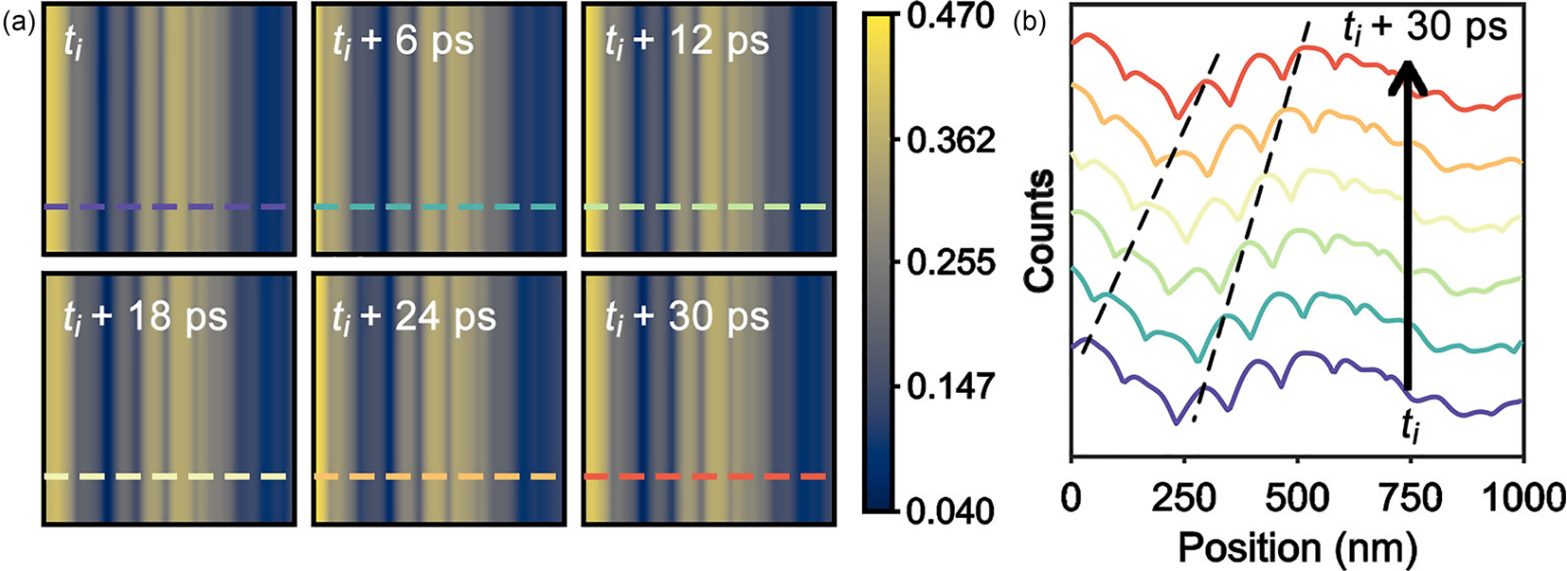
Simulated UEM bright-field images of transient, propagating distortions arising from excitation of the *S*_1_ Lamb mode using the dynamical multi-slice approach. The geometric arrangement (not shown) is the same as for the kinematic simulations. (a) Series of simulated UEM images at times *t* after excitation of the distortion, where *t_i_* > *t*_o_ is set as the initial response, and all other images are relative to *t_i_*. The colored dashed lines indicate the positions from which the line profiles in (b) were generated. The color bar is in units of counts normalized to vacuum. (b) Line profiles generated from the simulated images in (a). The colors correspond to the colored dashed lines in (a). The profiles are offset for clarity, and the propagating nature of the waves is illustrated with the black dashed lines, which connect the peaks of common wavefronts.

The approach taken for calculating the UEM bright-field images using the kinematic approximation (Fig. 2) was also used for the dynamical-scattering model, the results of which are summarized in Fig. 3. Qualitative visual inspection reveals that the contrast strength is significantly increased over that calculated using the kinematic approximation. Compared to a ratio of 2.4 for the kinematic approximation, the ratio of maximum to minimum normalized counts for the dynamical model is increased to 11.8 [Fig. 3(a)]. This is in better agreement with experimentally observed contrast strengths, suggesting that the model is a better quantitative descriptor of the contrast dynamics arising from propagating acoustic phonons observed with UEM bright-field imaging.^52,58^ While this result is expected, the model and approach described here will be useful for quantifying the basic properties of the propagating phonon modes giving rise to the imaged lattice distortions, including energies, strain states, and symmetries. Furthermore, this will be especially useful for determining how individual lattice discontinuities affect those properties, in addition to the propagation and dispersion behaviors readily accessible with UEM imaging. As mentioned above, the apparent dispersion behavior of the phonon velocities appeared in the dynamical simulations as well [Fig. 3(b)]. Again, the origins of this behavior are a product of the simulations only, as discussed above in the section describing the kinematic model. That is, though the single velocity and propagating nature of each wavefront is physically correct and emerges from the constraints placed upon the phonon behaviors, the velocity dispersion is not physically meaningful here, despite apparent agreement with experiment.^58^

While the dynamical scattering simulations return contrast strengths that are more physically meaningful and that better match experimental observations, it is important to restate that the model used here does not capture other potential contrast-forming mechanisms that may contribute to the observed behaviors. That is, while the simulations and model presented here do capture the effects of the complex strain profiles associated with Lamb-mode acoustic phonons, it is nevertheless still based solely on a pure-strain distortion and an underlying background absorption. Indeed, other contributing sources may exist for strongly photoexcited semiconductors. For example, strong photoexcitation leading to the generation of significant charge-carrier densities and associated plasma waves can produce an associated acoustoelectric effect, where lattice strain waves can effectively sweep carriers along the phonon wavefronts.^17,85–91^ Owing to the dependence of the Howie-Whelan formalism on the Fourier coefficients of the electrostatic potentials in the lattice,^64^ it would therefore be interesting to consider the effect fs photoexcitation of large carrier densities in semiconductors has on the transient responses imaged with UEM.

## A NOTE ON THE INFLUENCE OF CHANGES IN SPECIMEN ORIENTATION

The general manner in which acoustic-phonon dynamics are observable with UEM imaging—a local modulation of the lattice with respect to ***k_i_***—suggests that certain conditions may exist where strain-waves may be present but not observed. That is, if the energies and the resulting strains associated with the low-frequency, propagating phonons are such that relatively slight changes in lattice orientation occur (i.e., modulation of *s***_*g*_** is slight), then a potential prerequisite to observing the associated contrast dynamics would be orientation at least nearly along a crystallographic zone axis. Indeed, knowing the magnitude of lattice-orientation modulation by the strain wave, along with specimen geometry and thickness, would enable estimation of the allowable deviation from ideal Bragg scattering while still observing coherent contrast-wave dynamics. It is important to note that this idea is the same as for static, conventional TEM bright-field imaging and parallel-beam diffraction conditions, as well as ultrafast electron diffraction measurements; changes in the degree to which the lattice is oriented relative to ***k_i_*** across the field of view (of which there can be several sources) modulate intensities of the Bragg beams and, thus, the diffraction-contrast strengths.^92–94^ Accordingly, identification and quantification of these effects are needed for accurate assignment and characterization of the underlying ultrafast physical mechanisms.

**FIG. 4.**
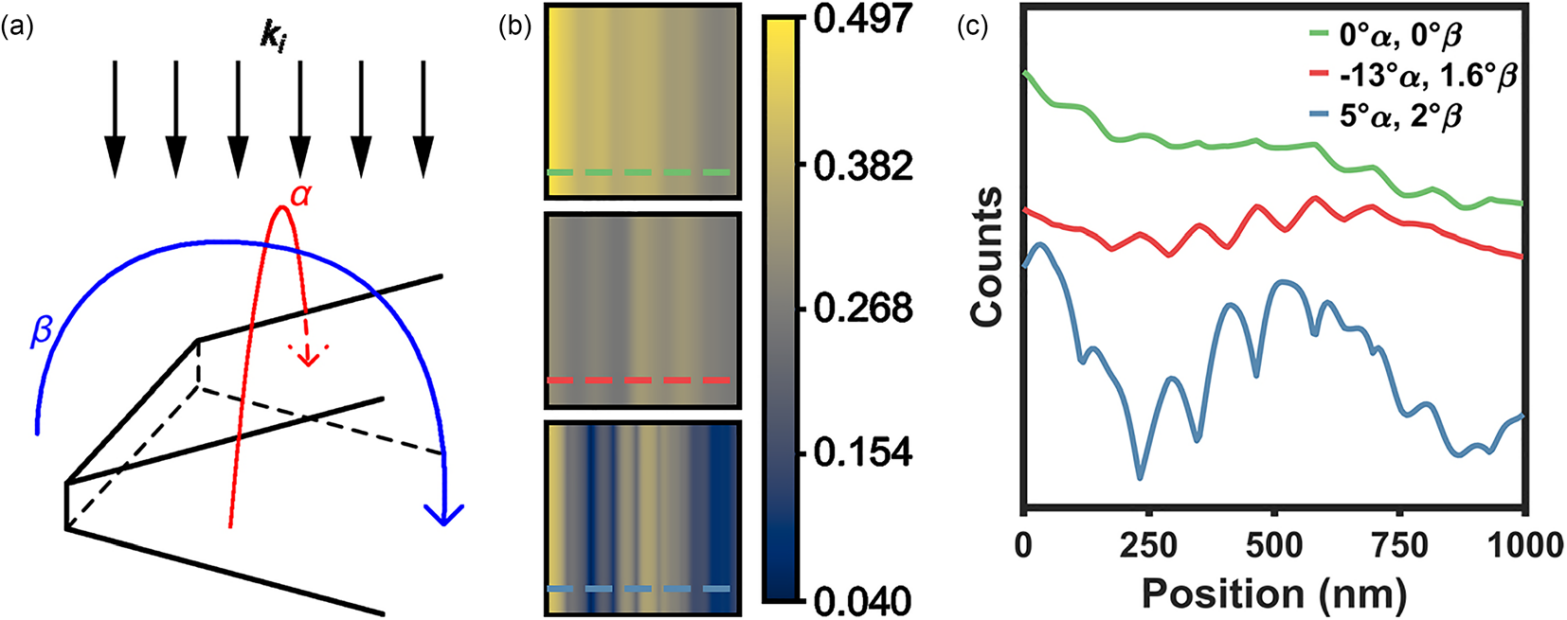
Effects of global specimen tilting on the simulated strain-wave bright-field contrast. (a) Schematic illustrating the simulation geometric arrangement with respect to the *α* (red) and *β* (blue) tilt directions. The incident electron wave vector, ***k***_***i***_, and the parallel-beam condition (series of parallel arrows) are also shown. (b) Simulated UEM bright-field images, all at the same time *t_i_* > *t*_o_ but with the specimen at different orientations with respect to ***k***_***i***_. Top panel: *α* and *β* = 0°. Middle panel: *α* = −13°, *β* = 1.6°. Bottom panel: *α* = 5°, *β* = 2° (as in Figs. 2 and 3). The colored dashed lines indicate the positions from which the line profiles in (c) were generated. The color bar is in units of counts normalized to vacuum. (c) Line profiles generated from the simulated images in (b). The colors correspond to the colored dashed lines in (b), and the profiles are offset for clarity.

The effects of varying specimen orientation with respect to ***k_i_*** on the observed contrast strengths from the dynamical simulations described above are summarized in Fig. 4. Here, two different *α* and *β* tilt-angle orientations were compared to the orientation shown in Figs. 2 and 3, which was found to be the optimum orientation for maximizing the contrast strength for the chosen conditions (*α* = 5° and *β* = 2°). As expected, the contrast strength of the bands arising from the excitation and propagation of strain waves across the field of view is significantly modulated when the global orientation of the specimen with respect to ***k_i_*** is changed. Indeed, orienting the specimen directly along the [*110*] zone axis at *α* = *β* = 0° (i.e., precisely parallel to the specimen upper-surface normal, where the upper surface is closest to the incident beam) reduces the ratio of maximum to minimum image counts from 11.8 to 1.8 [Fig. 4(b)].

As mentioned above, the results in Fig. 4 have implications worth discussing. First, the tilting simulations further illustrate why diffraction contrast is a useful tool for observing structural dynamics with UEM bright-field imaging, especially in the weak-excitation regime.^63,95^ Owing to significant intensity variation along the relrods, even slight movement of the reciprocal lattice with respect to a fixed Ewald sphere—when on or near the zone axis—will produce a measureable change in image and diffraction intensity.^64,94^ This sensitivity, however, may also pose a challenge to interpretation and assignment of dynamics when monitoring Bragg-spot intensities as a function of time. Second, the results indicate that phonon dynamics may be observable only in specimen regions that are initially oriented along (or nearly along) a zone axis, where overlap of the reciprocal lattice with the Ewald sphere is significant. This suggests that the absence of observable contrast-wave dynamics in certain specimen regions does not necessarily mean that the lattice is not in motion at those positions. Instead, it could simply indicate that those regions are not oriented near a zone axis. In such a configuration, relrod motion would not result in Ewald-sphere intersection. Third, accurate quantification of strain-wave dynamics (e.g., energies, strain states, and symmetries) from UEM imaging thus requires precise knowledge of the orientation of the local crystal lattice with respect to ***k_i_***, as well as the local and global motion of the lattice upon photoexcitation. Indeed, this last point is explicitly illustrated in Fig. 4.

## SUMMARY AND OUTLOOK

In summary, we have conducted both kinematical and dynamical simulations of UEM bright-field images of coherent Lamb-mode guided waves propagating through a pristine single-crystal Ge wedge specimen. By applying conventional, static-imaging approaches to strain fields calculated from a continuum mechanics model, we find that strain states on the order of 1% and less are readily observable as coherent contrast bands in the UEM bright-field images. Furthermore, by incorporating dynamical effects into the model using the Howie–Whelan equations and the approximations described by De Graef, we find that the contrast strength is increased by nearly an order of magnitude as compared to the kinematic approximation. Finally, we demonstrate the impact of global specimen motion on the observed contrast strengths arising from coherent acoustic phonons, and we discuss the implications for such an effect on ultrafast electron imaging. Looking forward, we anticipate that further refinement and increased sophistication of the work presented here will enable quantification of key aspects of photoexcited strain-wave phenomena in materials via fs electron imaging using UEM. This will not only allow for high-resolution probing of transient, nanoscale constitutive material properties, but it will also provide a means for developing a better understanding of the quantitative influence of spatially ill-defined variations in bonding and structure on those properties.

## AUTHORS' CONTRIBUTIONS

D.X.D.'s contributions were model analysis, simulation scripting, investigation, validation, visualization, writing—original draft, writing—review, and editing. D.J.F.'s contributions were conceptualization, funding acquisition, model analysis, project direction, resources, supervision, visualization, writing—original draft, writing—review, and editing.

## SUPPLEMENTARY MATERIAL

See the supplementary material for four supplementary videos. Supplementary video 1: Simulated bright-field UEM video using the kinematical approximation with the specimen oriented at *α* = 5° and *β* = 2°. Supplementary video 2: Simulated bright-field UEM video using dynamical-scattering theory with the specimen oriented at *α* = 5° and *β* = 2°. Supplementary video 3: Simulated bright-field UEM video using dynamical-scattering theory with the specimen oriented at *α* = 0° and *β* = 0°. Supplementary video 4: Simulated bright-field UEM video using dynamical-scattering theory with the specimen oriented at *α* = −13° and *β* = 1.6°.
